# The undervalued potential of positional therapy in position**-**dependent snoring and obstructive sleep apnea**—**a review of the literature

**DOI:** 10.1007/s11325-012-0683-5

**Published:** 2012-03-24

**Authors:** M. J. L. Ravesloot, J. P. van Maanen, L. Dun, N. de Vries

**Affiliations:** Department of Otolaryngology/Head Neck Surgery, Jan Tooropstraat 164, Sint Lucas Andreas Ziekenhuis, 1061 AE Amsterdam, the Netherlands

**Keywords:** Sleep apnea, Obstructive, Review, Sleeping position, Positional therapy

## Abstract

**Purpose:**

Research during the past 10–20 years shows that positional therapy (PT) has a significant influence on the apnea–hypopnea index. These studies are predominantly performed as case series on a comparably small number of patients. Still, results have not found their way into the daily diagnostic and treatment routine. An average of 56 % of patients with obstructive sleep apnea (OSA) have position-dependent OSA (POSA), commonly defined as a *difference of 50 % or more in apnea index between supine and non-supine positions*. A great deal could be gained in treating patients with POSA with PT. The aim of this paper was to perform a thorough review of the literature on positional sleep apnea and its therapy.

**Methods:**

A broad search strategy was run electronically in the MEDLINE and EMBASE databases using synonyms for position and sleep apnea.

**Results:**

Sixteen studies were found which examined the effect of PT on OSA. In this literature review, we discuss the various techniques, results, and compliance rates.

**Conclusion:**

Long-term compliance for PT remains an issue, and although remarkable results have been shown using innovative treatment concepts for PT, there is room for both technical improvement of the devices and for further research.

## Introduction

Snoring and obstructive sleep apnea (OSA) are the most prevalent sleep-disordered breathing problems. OSA affects 2–26 % of the general population, depending on gender, age, and definition of the criteria used [1, 2]. OSA is associated with significant morbidity, such as excessive daytime sleepiness, socially unacceptable snoring, and impaired quality of life. Patients are at higher risk of developing cardiovascular diseases [3, 4]. If the apnea–hypopnea index (AHI) is >40, the risk of being involved in a traffic accident increases [5, 6].

Adequate treatment is of key importance. Continuous positive airway pressure (CPAP) is regarded as the gold standard treatment of OSA, with mandibular advancement device (MAD) therapy or surgery in reserve for CPAP failures [7]. Unfortunately, 29–83 % of patients prescribed CPAP are non-adherent and use their CPAP less than 4 h per night [8, 9]. In cases of CPAP failure, treatment remains indicated. MADs and a variety of surgical interventions are then available [10–15]. All these treatment modalities have their specific downsides.

Conservative treatment of OSA can be just as crucial: lifestyle alterations such as weight reduction, abstinence of alcohol and sedatives, and avoidance of supine sleeping position, where appropriate. Significant improvement and even remission was recorded in obese patients diagnosed with OSA undergoing BS [16]. The latter should be considered as a treatment option for patients with severe OSA and obesity alongside CPAP.

A number of papers have been published on the role of supine position on OSA and methods to avoid supine position. In 1948, in a “plea for more serious consideration of snoring,” Robin [17] stated that “sleeping on one’s back is considered a common cause of snoring.” It is likely that spouses of (apneic or non-apneic) snorers were the first to identify the role of body position on the severity of the snoring or apnea of their bed partner. In 1984, Chest published a letter written by a patient’s wife [18]. She had cured her husband’s sleep apnea snoring problem by “having sewn a pocket into the back of a T-shirt and having inserted a hollow, lightweight plastic ball to prevent her husband sleeping on his back.” During the American War of Independence (1775–1783) and later during World War I (1914–1918), soldiers were advised to wear their rucksacks (filled with a bulky mass) while sleeping in order to avoid sleeping on their backs and reduce snoring so as to avoid making their position known to the enemy.

PT, in whichever form, has been found to have a significant influence on snoring and OSA severity [7]. Still, results have not found their way into the daily OSA diagnostic and treatment routine, even though approximately 56 % of patients with OSA have position-dependent OSA [19–22]. Why is PT unfashionable?

By means of a thorough review of the literature on positional sleep apnea and its therapy, this study aims to provide an overview of the various PT techniques and their success and compliance rates.

## Method

A broad search strategy was run electronically in the MEDLINE and EMBASE databases on 5 October 2011 by one researcher (M.R.): (“Position” OR “position dependent“ OR “positional” OR “posture”) AND (“apnea” OR “apnoea” OR “OSA” OR “OSAS” OR snor*). In addition, the reference lists of included articles were screened for additional relevant citations. Studies were evaluated according to the Oxford Centre for Evidence-Based Medicine levels of evidence (Table 1).

**Table 1:** Oxford Centre for Evidence-Based Medicine levels of evidence

Level	Therapy
1a	Systematic review of randomized controlled trials
1b	Individual randomized controlled trial
2a	Systematic review of cohort studies
2b	Individual cohort study
2c	“Outcomes research”
3a	Systematic review of case–control studies
3b	Individual case–control study
4	Case series (with or without comparison)
5	Expert opinion

## Diagnosis

### Current definition of OSA and POSA

The recommended objective diagnostic criteria for OSA include an AHI of 5 or more and evidence of daytime sleepiness. The AHI is defined as the mean number of apneas and hypopneas per hour during sleep, an apnea is a period of 10 s or more with a reduction of oronasal airflow of >90 %. A hypopnea is defined as an episode of more than 30 % airflow reduction of the baseline (calculated from the preceding period of 100 s) during at least 10 s. Suggested AHI thresholds are 5, 15, and 30 events per hour for mild, moderate, and severe levels of OSA, respectively [23, 24].

Cartwright [25] was the first to describe the arbitrary cutoff point of a *difference of 50 % or more in apnea index between supine and non-supine positions*. This is the most common definition for positional obstructive sleep apnea (POSA) used today, but many question Cartwright’s criteria and apply adapted versions.

Both Mador’s and Permut’s groups defined POSA as follows: “an AHI of fewer than 5 events per hour while in the non-supine position as well as a decrease in the AHI by more than 50 %” [26, 27]. In 1998, Marklund et al. [28] defined supine-dependent sleep apnea as follows: a supine apnea–hypopnea index ≥10, together with a lateral apnea–hypopnea index <10. In the study of Bignold et al. [29], when patients met the following criteria, they were deemed position-dependent: “overall AHI ≥15/h, supine AHI ≥ twice the non-supine AHI; ≥20 min of sleep in supine and non-supine postures and non-supine AHI <15.”

### Sleep study with sleep position assessment and separate assessment for head and trunk

Sleep studies exist in many varieties, from very simple to very detailed. In order to determine whether a patient is position-dependent, it is clear that assessment of the sleep position is mandatory. In every OSA patient, the role of sleep position should be investigated. The role of sleep studies without sleep positional recording is therefore very limited. Ideally, both the mean AHI as well as the separate AHIs in supine, left, right, and prone sleep positions should be recorded. In the hypnogram, this is reflected by a clustering of respiratory events correlated with the change in body position.

Commonly, position sensors are attached to the elastic bands around either the chest or abdomen. Our group recently confirmed the hypothesis that the occurrence of OSA may also be dependent on the position of the head [30]. Study subjects underwent overnight polysomnography with two position sensors: one on the trunk and one in the mid-forehead. Overnight results based on the two different sensor positions show that the AHI calculated over the total sleep period with the head lying supine was frequently higher than the AHI calculated over the total period with the trunk in supine position. Over the time period when lying on the back with the head also supine, the AHI was significantly higher than during the time period with the head turned sideways. The use of dual position sensors could have major clinical and research implications [31]. In patients with a suspicion of position-dependent OSA, sleep recording with dual position sensors placed on both trunk and head should be considered.

## Prevalence of position-dependent OSA and snoring

A considerable amount of literature exits on the role of sleep position in OSA [19–22, 25, 26, 30–37]. In studies from Israel and the Netherlands, a remarkable steady 56 % of patients with OSA have a difference of 50 % or more in the apnea index between the supine and non-supine positions [19–22]. An additional 30 % of patients have a higher AHI in the supine position than in the other positions, but not twice as high. On average, patients with POSA have a lower BMI and are younger than non-positional OSA patients [19, 22].

Reports have suggested that snoring is aggravated by a supine sleeping position [25, 38]. Nakano et al. [39] described the effect of position on snoring in 21 non-apneic (AHI < 15) and 51 apneic patients (AHI > 15). They conclude that “Snoring time as well as snoring intensity was lower in the lateral position than in the supine position in the non-apneic patients whilst in the apneic group neither snoring time nor intensity had statistical differences.” In non-apneic patients, the snoring time was 17.5 % and 6.4 % and the intensity 101.6 and 98.3 dB in the supine and non-supine positions, respectively. In the apneic group, the snoring time was 16.9 % and 15.4 % and the intensity 102.9 and 103.5 dB in the supine and non-supine positions, respectively.

Choi et al. [40] defined a position-dependent snorer as “one who has a greater than 50 % reduction of snoring rate in the lateral position compared to that in the supine position.” To our best knowledge, the prevalence of position-dependent snoring is yet to be reported.

## Positional therapy

### Positional therapy

PT can be defined as *preventing patients to sleep in the worst sleeping position*. The worst sleeping position is usually, but not always, the supine position [35]. Various techniques are described to prevent patients from assuming the supine position such as positional alarms, verbal instructions, tennis balls (TBT), vests, “shark fins,” or special pillows [7, 27, 29, 40–53].

#### The effect of positional therapy on snoring

##### Rationale

In 1948, Robin [17] stated that “many persons snore only when on their backs” and suggested that on some occasions, sewing a cotton reel into the back of a pyjama can be effective, albeit rather uncomfortable. The effect of PT on snoring can be measured from various angles: intensity (decibels), frequency (snores/hour), snoring rate (% TST), or duration (seconds or milliseconds).

##### Overview of evidence

Two studies specifically studied the effect of PT on snoring. In five studies evaluating the effect of PT on POSA, the result on snoring was also mentioned.

Braver and Block [54] reported that PT (foam rubber wedges both behind and in front of subject) was not effective in reducing snoring in 20 patients. The number of snores remained 356/hour both with and without PT.

Choi et al. [40] evaluated the efficacy of PT (vest with two inflatable chambers) to treat snoring in 17 positional-dependent snorers, defined as one who has a >50 % reduction of snoring rate in the lateral position compared with that in the supine position. The snoring rate decreased from 36.7 % to 15.7 % without subjective or objective adverse effects.

Maurer et al. [41] found an overall decrease in snoring time from 180 to 110 min in 12 apneic patients treated with PT (vest with semi-rigid foam in its dorsal part), but an increase was observed in 30 % of the patients. A statistically significant decrease in snoring was reported by Zuberi et al. [42] in 22 patients with POSA treated with PT (triangular pillow), whilst Wenzel et al. [43] reported a decrease in snoring rate from 15.4 % to 9.8 % in 14 patients with POSA treated with PT (vest). Loord and Hultcrantz [44] reported that half of the patients (*n* = 18) treated with PT (soft vest attached to a board with pillow) snored more frequently; specifically, six snored less frequently, nine snored more frequently, and for two, there was no difference. A recent study by Bignold et al. [29] reporting on the efficacy of the position monitoring and supine alarm device on 15 patients with supine-dependent OSA found no improvement in snoring. There was a trend for an overall reduction in snoring frequency, but this was not statistically significant. Furthermore, there was no difference in mean snore duration.

##### Conclusion

In non-apneic patients, snoring decreased when a patient adopted a non-supine position. In apneic patients, in the majority of studies, PT does not result in an improvement in snoring.

#### The effect of positional therapy on OSA

##### Overview of the evidence

A number of studies have examined the effect of PT on OSA [7]. Of the 23 relevant articles found, seven studies were excluded from the overview. Two studies did not provide information on the effect of PT on OSA parameters and were omitted from the overview [46, 47]. Five studies evaluated the effect on OSA of an array of devices resulting in an elevated posture and head extension [55–59]. As these devices did not prevent the patient from assuming the supine position, we did not include these studies in our review. An overview of the 16 included articles is presented in Table 2.

**Table 2:** Overview literature on PT

	Year	Design	Level of evidence	No.	BMI (kg/m^2^)	PT method	Mean AHI without PT	Mean AHI with PT	Mean TST in supine position without PT (%)	Mean TST in supine position with PT (%)	AHI in supine position without PT	AHI in supine position with PT	AHI in non-supine position without PT	AHI in non-supine position with PT	Sleep efficiency without PT (%)	Sleep efficiency with PT (%)	Follow-up
Cartwright [45]	1985	Case series	4	10	30.6	Positional alarm	54.7	21.4	51.4	2.1	72.0	11.0	19.3	21.6	ND	ND	–
Kavey [32]	1985	Case series	4	2/4	24.5	Ball in sock on back	9.2^b,c^	3.8^b,c^	40.4^c^	8.8^c^	13.7^b,c^	5.1^b,c^	ND	ND	ND	ND	4–12 months
Kavey [32]	1985	Case series	4	2/4	26.5	Verbal instructions	40.8^b,c^	2.8^b,c^	92.1^c^	11.2^c^	42.4^b,c^	6.9^b,c^	ND	ND	ND	ND	3–4 months
Cartwright [46]	1991	Case series	4	15/60	ND	Positional alarm	33.3	20.8	141.1^f^	3.4^f^	62.5	32.9	9.7	21.7	ND	ND	8 weeks
Cartwright [46]	1991	Case series	4	15/60	ND	Verbal instructions	26.7	7.7	101.3^f^	16.5^f^	87.3	26.8	7.7	4.6	ND	ND	8 weeks
Braver [54]	1994	Randomized crossover trial	2b	20	36	Foam wedges	17.5	14.1	68	ND	ND	ND	ND	ND	ND	ND	–
Jokic [48]	1999	RCT	2b	13	30	Backpack with softball inside	17.9	9.5	25.6	1.7	63.8	ND	4.9	ND	ND	82	–
Maurer [41]	2003	Case series	4	12	26.5	Vest with semi-rigid foam on dorsal part	26.7	7.6	300^f^	63^f^	39.3	ND	5.5	ND	81	83	–
Zuberi [42]	2004	Case series	4	22	23–48^a^	Triangular pillow	23.5	11.1	ND	ND	ND	ND	ND	ND	ND	ND	–
Oksenberg [49]	2006	Case series	4	12	28.1	Tennis ball technique	46.5	17.5	79	12.3	57.0	44.4	11.6	13.8	80.9	78.9	2 months
Wenzel [43]	2007	Case series	4	14	28.1	Vest with semi-rigid foam on dorsal	31.3	13.8	72.2	2.1	ND	ND	ND	ND	86.3	78.9	–
Loord [44]	2007	Case series	4	18/23	ND	Soft vest attached to a board with pillow	21.8	14.3	ND	ND	50.4	ND	ND	ND	ND	ND	3 months
Skinner [51]	2009	Randomized crossover trial	2b	20	30.7	Thoracic anti-supine band (TASB)	22.7	12.0	34.4	6.3	59.6	37.8	4.7	10.3	ND	ND	1 month
Permut [27]	2010	Randomized crossover trial	2b	38	31	Vest with semi-rigid foam on dorsal part	11^d^	2^d^	40^d^	0^d^	31^d^	ND	2^d^	ND	89^d^	88^d^	–
Choi [40]	2011	Case series	4	17	ND	Vest with inflatable chambers	7.7	4.8	67.1	25	ND	ND	ND	ND	89.8	87.6	
Svatikova [52]	2011	Randomized, controlled, crossover trial	2b	18	29	Triangular pillow	39^d^	27^d^	39	8	49^d^	51^d^	27^d^	27^d^	ND	ND	–
Bignold [29]	2011	Randomized, crossover trial	2b	15	28.8	Position monitoring and supine alarm device	24.1	–^e^	36.4	ND	51.3	ND	9.7	ND	81.4	ND	–
Maanen [53]	2011	Randomized, controlled, single-blind, crossover trial	2b	30	27.7	Neck-worn vibrating apparatus	27.7	12.8	40	19	59.7	12.5	6.7	11.2	91.9	88.3	–

*AI* apnea index, *AHI* apnea–hypopnea index, *BMI* body mass index, *PT* positional therapy, *TST* total sleep time, *ND* not defined

^a^Range

^b^Apnea index

^c^Initial diagnosis based on two consecutive PSGs. After initial diagnosis, patients studied for an additional one or two nights between 4 months and 3 years later, during which time they avoided sleeping in the supine position

^d^Median

^e^AHI reduction in the order of 45 % with active treatment estimated from nasal cannula/oximetry from home-sleep studies

^f^In minutes

Various techniques are described to prevent patients from assuming the supine position, such as an upright sleep posture, positional alarms, verbal instructions, TBT, vests, “shark fins,” or special pillows [7, 27, 29, 40–53].

In an attempt to decrease discomfort and improve compliance, our group developed a new treatment concept: a small neck-worn vibrating device which prevents patients from applying a supine sleeping position [53]. When wearing the device, adopting a supine position triggers a vibration which increases in intensity until a new position is adopted, without significantly reducing total sleep time or disrupting sleep. Thirty patients with positional sleep apnea were included in a pilot study. No side effects were reported. The mean AHI dropped from 27.7 ± 2.4 to 12.8 ± 2.2. Seven patients developed an overall AHI below 5 when using the device in ON modus. Although the results are encouraging, several items remain to be addressed with this device, and there is room for improvement. The long-term effect remains to be studied.

Bignold et al. [29] evaluated the efficacy of a similar device in 15 patients fulfilling the following criteria: overall AHI ≥15 per hour, supine AHI twice or greater than the non-supine AHI; ≥20 min of sleep in supine and non-supine postures and non-supine AHI < 15. Subjects were assigned to receive the active PT or the inactive PT in a random order for a week followed by a 1-week washout before commencing the alternative treatment. The device consists of a position-monitoring and supine alarm device fastened to the chest. The mean baseline AHI (24.1) was reduced in the order of 45 % with active treatment.

Three publications studied the effect of PT compared to CPAP in a randomized crossover study setup. Jokic et al. [48] included 13 patients who were randomized to 2 weeks of treatment with nasal continuous positive pressure (nCPAP) or PT (backpack with softball) followed by a crossover to the other modality. They found “PT to be highly effective in reducing time spent in a supine position.” And although both treatment modalities were found to improve OSA severity, nCPAP was found to be more effective in reducing the AHI (17.9 to 3.4 on nCPAP, to 9.5 with PT).

Skinner et al. [51] included 20 patients in a randomized crossover comparing the efficacy of a thoracic anti-supine band (TASB) with nCPAP. Subjects were randomly assigned to receive the TASB or nCPAP for the first month followed by a 1-week washout before commencing the alternative treatment*.* The baseline AHI was 22.7 and was decreased to 12.0 with TASB and 4.9 with nCPAP. A successful treatment outcome was defined as an AHI < 10, which was achieved in 13 of 18 subjects when using TASB and in 16 of 18 subjects when using nCPAP. Once again, they found that the self-reported compliance was significantly better with TASB than with nCPAP. Nineteen of 20 patients reported a 7-h nightly use of the TASB, while only 9 of 20 patients managed to use their nCPAP at least 4 h per night.

The recent study by Permut et al. [27] showed that PT (a bulky mass strapped to the back) was equal to CPAP in normalizing the AHI in patients with a mild to moderate POSA. Only patients with a non-supine AHI of <5 were included. The long-term effect was not reported.

##### Conclusion

All studies report a positive effect of PT on the AHI. PT compliance is better than CPAP compliance, but the latter is a more effective treatment.

### Positional therapy compliance

Even the most effective medical devices are only effective when they are used. Both CPAP and, to a lesser extent, MAD therapy are hampered by compliance issues [60–62].

#### Overview of the evidence

Skinner et al. [51] included 20 patients in a randomized crossover comparing the efficacy of TASB with nCPAP. Subjects were randomly assigned to receive TASB or nCPAP for the first month followed by a 1-week washout before commencing the alternative treatment. The self-reported compliance was significantly better with TASB than with nCPAP. Nineteen of 20 patients reported a 7-h nightly use with the TASB. In contrast, only 9 of 20 subjects met the 4-h per night CPAP compliance criteria.

Next to the efficacy study of PT (vest with semi-rigid foam on dorsal part) by Wenzel et al. [43], the group contacted the patients approximately 13.7 months later by telephone to assess PT compliance. Only 4 of the 14 patients were still using PT (on average for 7.3 h and 6.4 nights); their ESS was reduced from 8.5 to 6.5. The remaining 10 patients had stopped using PT due to the following reasons: discomfort and tightness of the vest, frequent awakenings, restless sleep, increased sweating during the night, and prevention of preferred sleeping position.

Oksenberg et al. [49] assessed the use of PT (TBT) during a 6-month period in 78 consecutive POSA patients. Of the 50 patients who returned the questionnaire, 38 % were still using PT, 24 % no longer used PT as they claimed to have learned to avoid the supine position, and 38 % no longer used PT, but had not learned to avoid the supine position.

Bignold et al. [50] studied the compliance of 67 patients, who had been prescribed PT (TBT) 2.5 ± 1 years earlier, using a follow-up questionnaire. Six percent were still using PT, 13.4 % no longer used PT as they claimed to have learned to avoid the supine position, and a staggering 80.6 % no longer used PT, but had not learned to avoid the supine position. Reasons to abort the PT included ineffectiveness, backache, discomfort, and no improvement in sleep quality or daytime alertness.

Of the nine patients randomized to PT (triangular pillow), in a study performed by Svatikova et al. [52], 3 months post-stroke, the self-reported adherence was: 3 (33 %) all nights, 1 (11 %) most nights, 2 (22 %) some nights, and 3 (33 %) no nights.

In a second study performed by Bignold et al. [29], patients were assigned with PT for 3 weeks (a position monitoring device and supine alarm device). The device was active for one of the 3 weeks. Patients used the device 85 % of nights over the full 3 weeks, with an average of 6.8 h of use per night.

It has been suggested that patients may learn to avoid the supine position following PT and therefore do not need to use PT on a regular basis [45]. Others may need PT either periodically to reinforce training or consistently.

#### Conclusion

Ineffectiveness, backache, discomfort, and no improvement in sleep quality or daytime alertness have been responsible for poor compliance and the subsequent disappointing long-term results of PT.

### Can the effect of positional therapy be predicted from the sleep study?

Many different forms of sleep study are available, some simple and some more extensive. Some take sleep position into account; others do not. Most provide information on sleep position, time spent per position, and the AHI distribution per position. Some polysomnographies (PSGs) calculate the non-supine AHI; if not, the following formula can be used:

$$ \frac{{\left( {{\text{AH}}{{\text{I}}_{\text{prone}}} \times {\text{TS}}{{\text{T}}_{\text{prone}}}} \right) + \left( {{\text{AH}}{{\text{I}}_{\text{left}}} \times {\text{TS}}{{\text{T}}_{\text{left}}}} \right) + \left( {{\text{AH}}{{\text{I}}_{\text{right}}} \times {\text{TS}}{{\text{T}}_{\text{right}}}} \right)}}{{{\text{TS}}{{\text{T}}_{\text{prone}}} + {\text{TS}}{{\text{T}}_{\text{left}}} + {\text{TS}}{{\text{T}}_{\text{right}}}}} $$

It remains to be studied what the predictive value of the non-supine AHI is. Can it be used to indicate when PT may be successful or to measure the expected effect of PT? Both Mador’s and Permut’s groups only included patients with an AHI of fewer than five events per hour while in the non-supine position [26, 27].

### Sleep position and positional therapy in combination with sleep surgery

#### Rationale

As early as 1948, Robin [17] wrote: “sleeping on one’s back is considered a common cause of snoring, as the tongue falls back more readily.” He reasoned that “by changing the position of the head the tongue will be prevented from falling back,” Harper and Sauerland [63] suggested that “when sleep apnea patients sleep in supine position, the tongue tends to fall backward against the pharyngeal wall, due to gravity.” Our group recently reported that visualization of a base of tongue obstruction or epiglottis obstruction during DISE was more common in patients with POSA in comparison to patients with OSA (*p* = 0.058) [64].

These results suggest a trend: patients with POSA may require base of tongue level surgery more often than patients without positional dependence. Is this an overlooked cause of surgery failures?

#### Overview of the evidence

To our best knowledge, three papers have been written on the effect of sleep position on treatment outcomes of sleep surgery, the uvulopalatopharyngoplasty (UPPP).

Katsantonis et al. [65] studied the effect of UPPP on sleep posture and differences in UPPP results in various sleep positions in a small series of 17 patients. They found that following UPPP, the AHI significantly improved in the lateral position. They also found that during sleep in a supine position, the AHI did not show a significant improvement. They conclude that “UPPP enhances the position effect on OSA because it readily eliminates obstructive events in the lateral sleep position.” In other words, the difference in AHI in the supine and non-supine positions is more pronounced postoperatively. They are of the opinion “that additional PT could significantly improve response to treatment with UPPP.”

Lee et al. [66] studied the effect of sleep position on surgical outcomes as well. They studied 69 consecutive patients who underwent a UPPP. After categorizing the patients into four groups according to the change in AHI after surgery, they found that the failure group had a higher proportion of supine position dependency than any other group.

In a second paper published by the same group, the results show that UPPP is a successful treatment for obstructive events occurring in the lateral sleep position, especially in patients without positional dependency [67]. The suggestion is made that “patients who have become position dependent may benefit from PT after UPPP.”

A Korean study evaluated the changes of sleep positions before and after pharyngeal (UPPP or uvulopalatal flap or tonsillectomy) and/or nasal surgery (endoscopic sinus surgery and/or septoplasty and/or turbinoplasty) in 52 OSA patients with no response to surgery (*n* = 25) and with response to surgery (*n* = 28) [68]. Response was defined as a >50 % decrease in postoperative AHI. They concluded that “the frequency of positional changes was significantly decreased with the improvement of respiratory disturbances and arousals in the response group after surgery.”

#### Conclusion

All three papers conclude that UPPP is most successful in decreasing the AHI in the lateral position. In the supine position, following UPPP, the AHI shows no significant improvement. As the difference in AHI in the supine and non-supine positions is more pronounced postoperatively, UPPP enhances the position effect on OSA; therefore, additional PT could significantly improve response to treatment.

### Sleep position and positional therapy in combination with an oral device

#### Overview of the evidence

Four papers were found which studied the treatment outcome of MAD therapy specifically in positional and non-positional OSA patients.

Cartwright [69] investigated factors associated with the effectiveness of an MAD on OSA in 16 male patients. Patients with position-dependent OSA were more responsive to MAD therapy than patients with non-position-dependent OSA*.* The presence of an increased severity of apneas in the supine posture was the strongest predictor of success.

Yoshida [70] studied the effect of an MAD in 72 patients according to sleep position. Forty-four patients exhibited apneas *most frequently* in the supine position, 15 in the lateral position, and 13 in the prone position. The baseline AHI was significantly lower in the prone group than in the lateral group or the supine group. In the supine group, the treatment was successful (defined as an AHI < 10) in 61.4 % of the patients, none in the lateral group, and 84.6 % in the prone group. Yoshida concluded that the effectiveness of an oral appliance is greatly influenced by sleep posture.

Marklund et al. [28], in a small series, found treatment success to be related to supine-dependent sleep apnea. Supine-dependent sleep apnea was defined when the supine apnea–hypopnea index was ≥10, in combination with a lateral apnea–hypopnea index of <10. In 12 patients with supine-dependent sleep apnea, an MAD reduced the supine apnea–hypopnea index from a median of 41 to 5.9. In 14 patients with non-supine-dependent sleep apnea, the treatment reduced the supine apnea–hypopnea index from 44 to 21 and the lateral apnea–hypopnea index from 21 to 4.5. The adjusted odds ratio for a successful apnea reduction to an apnea–hypopnea index of <10 in both the supine and the lateral positions was 30 for supine-dependent sleep apnea.

Chung et al. [71] studied 72 consecutive patients (42 patients with and 30 without position-dependent sleep apnea when applying Cartwright’s POSA criteria) who underwent a sleep study before and after the insertion of an MAD. They found that “patients with positional OSA had substantially better treatment outcomes than patients with non-positional OSA.” Both the decrease in overall and supine AHI was significantly greater in the positional OSA group.

The role of combination therapy—MAD with PT—remains to be further elucidated, but seems promising. Cartwright [46] showed that the “combined effect of PT and a tongue retaining device was better than one of the treatment modalities alone.” Sixty patients with an AHI of at least 12.5 were randomly assigned to either (1) MAD, (2) PT (positional alarm), or (3) combination therapy (MAD and PT). The AHI was reduced from 27.4 to 11.4 in group 1, from 33.3 to 20.8 in group 2, and from 30.7 to 7.9 in group 3.

#### Conclusion

In brief, they all conclude that MADs are more effective in patients with positional OSA than in patients without positional OSA. The role of combination therapy remains to be further elucidated.

### Sleep position and positional therapy in combination with CPAP

As mentioned, CPAP compliance is often poor. One of the many reasons for CPAP failure and non-compliance is high CPAP pressure.

#### Overview of the literature

In a retrospective study by Pevernagie and Shephard [72], patients diagnosed with OSAS returned for a second overnight sleep study, during which nCPAP was titrated up to a level that eliminated SDB events and snoring in the supine position. Thirty-one patients who had sufficient sleep time in NREM and REM sleep in both supine and non-supine sleep postures were included. They found that “patients with positional sleep apnea required less positive airway pressure, than non-positionals, as well as a tendency to avoid sleeping on the back in direct proportion to the severity of their OSA in that position.”

In contrast, in a small-scale study, Sériès and Marc [73] concluded that CPAP compliance improved with auto-CPAP therapy in patients with sleep stage- and/or body position-dependent nocturnal breathing disorders compared to fixed CPAP. The effective pressure/time index was significantly lower in sleep stage- and body position-dependent patients treated with fixed CPAP than in the other patients.

Oksenberg et al. [74] concluded in a retrospective study of 83 consecutive patients undergoing nCPAP titration that the optimal nCPAP level was significantly higher in the supine position than it was in the lateral position.

Body position and sleep stage have been shown to significantly influence the positive pressure level needed to treat obstructive breathing abnormalities. Pressure level requirements may vary over time due to several factors such as weight loss or gain, medication and alcohol use, nasal congestion, changes in jaw position (due to an MAD for example), duration of CPAP therapy (CPAP is thought to play a role in reducing edema resulting from snoring-associated vibration and apnea-induced suction of the upper airway), the cyclic alternating pattern of sleep stages, or body position [60, 72–74].

#### Conclusion

Most studies suggest that patients’ positive pressure needed in the supine position is greater than that needed in the non-supine position. Therefore, patients benefit from auto-CPAP, with a consequent increase in compliance.

PT could theoretically be of value. The treatment of OSA is a stepwise approach. If a patient with supine-dependent OSA can avoid the supine position, the consequent decrease in AHI and positive pressure requirements results in less aggressive treatment, improving tolerance and compliance [75].

### Sleep position and nasal expiratory device

One study was found to have examined the effect of sleep position on the efficacy of the novel treatment: the nasal expiratory resistor device (nEPAP) [76]. Twenty subjects with OSA were included in the study who underwent PSG while wearing the therapy. The results suggest that patients with position-dependent SDB (defined as a supine AHI greater than the lateral AHI) were more likely to have an acceptable therapeutic response to nEPAP, although the results did not reach statistical significance.

## Discussion and future perspectives

Unfortunately, research on the effect of PT on POSA lacks good clinical trials, a miss in OSA research in general. Not all articles included in this paper specify definitions and cutoff values used to rule in OSA. In 1999, the American Academy of Sleep Medicine Task Force introduced evidence-based standardized scoring guidelines and cutoff values for OSA. Studies discussed in this paper may have applied different definitions, especially if performed before 1999 [23].

At present, evidence of PT effectiveness is based on small-scale case series and a few randomized trials. Little is known about the long-term compliance of PT and the actual ability of patients to learn to avoid the supine position following PT treatment.

There is room for technical improvement of the devices to reduce discomfort and consequent disruption of sleep architecture as to improve compliance.

POSA is commonly defined as a *difference of 50 % or more in apnea index between supine and non-supine positions*, but many question Cartwright’s criteria and apply adapted versions. Similar issues have faced CPAP compliance criteria and surgical and MAD success definitions [60]. CPAP therapy is regarded as successful if the AHI drops below 5 when CPAP is used. Current trends define compliance as 4 h per night as an average over *all* nights observed [61].

Surgical success was originally defined by Sher et al. [77] as an AHI reduction of at least 50 % and an AHI reduction to below 20**.**

Others have later proposed to tighten surgical success criteria to a postoperative AHI below 15 (regarded as “clinically relevant” OSA), below 10, and, recently, even below 5 [78]. Patients in whom the AHI is reduced by 20–50 % are classified as responders [12].

PT of OSA now commonly aims to arouse the patient when lying on the back so the subject rotates the body on his/her side to alleviate respiratory obstructions. In head position-aggravated trunk supine position-dependent OSA, it may be sufficient to stimulate the subject to rotate only the head sideways based on a position sensor monitoring the orientation of the head. It can be expected that this would have a much less profound negative effect on sleep quality.

## Conclusion

Research performed in the past 10–20 years show that PT has a significant influence on AHI. These studies are predominantly performed as case series on a comparably small number of patients. Still, results have not found their way into the daily diagnostic and treatment routine. An approximate 56 % of patients with OSA have position-dependent OSA commonly defined as a *difference of 50 % or more in apnea index between supine and non-supine positions*. A great deal is to be gained from treating patients with POSA with PT. PT, often simple and inexpensive, shows promise as a stand-alone treatment or as an additional measure to increase the success rate of other established treatment methods. Treating body position should receive more attention in the treatment of sleep apnea. Long-term compliance for PT remains an issue, and although remarkable results have been shown using innovative treatment concepts for PT, there is room for both technical improvement of the devices used and for further research.

## Abbreviations

AHI: Apnea–hypopnea index
AI: Apnea index
BMI: Body mass index
BS: Bariatric surgery
OSA: Obstructive sleep apnea
CPAP: Continuous positive pressure
DISE: Drug-induced sleep endoscopy
ESS: Epworth Sleepiness Scale
nCPAP: Nasal continuous positive pressure
MAD: Mandibular advancement device
POSA: Positional obstructive sleep apnea
PSG: Polysomnography
PT: Positional therapy
SaO_2_: Oxygen saturation
TBT: Tennis ball technique
UPPP: Uvulopalatopharyngoplasty
